# Depressive disorder at the household level: prevalence and correlates of depressive symptoms among household members

**DOI:** 10.1080/16549716.2023.2241808

**Published:** 2023-08-09

**Authors:** Kondwani Mpinga, Temusa Rukundo, Owen Mwale, Myrrah Kamwiyo, Limbani Thengo, Todd Ruderman, Beatrice Matanje, Fabien Munyaneza, Emilia Connolly, Kazione Kulisewa, Michael Udedi, Chiyembekezo Kachimanga, Luckson Dullie, Ryan McBain

**Affiliations:** a Monitoring and Evaluation, Medical Informatics, Information Technology and Research Department, Partners in Health, Neno, Malawi; b Clinical Department, Partners in Health, Neno, Malawi; c Pardee RAND Graduate School, RAND Corporation, Santa Monica, CA, USA; d College of Medicine, Kamuzu University of Health Sciences, Blantyre, Malawi; e Clinical Services, Ministry of Health, Lilongwe, Malawi; f Healthcare Delivery, RAND Corporation, Washington, DC, USA

**Keywords:** Depression, depressive disorder, social ecology, epidemiology, social determinants

## Abstract

**Background:**

Globally, an estimated five percent of adults have major depressive disorder. However, little is known about the relationship between these individuals’ depressive symptoms and their household members’ mental health and well-being.

**Objectives:**

We aimed to investigate the prevalence and predictors of depressive symptoms among adult household members of patients living with major depressive disorder in Neno District, Malawi.

**Methods:**

As part of a cluster randomized controlled trial providing depression care to adults with major depressive disorder, we conducted surveys with patients’ household members (*n* = 236) and inquired about their overall health, depressive symptoms, disability, and social support. We calculated prevalence rates of depressive disorder and conducted multivariable linear regression and multivariable logistic regression analyses to assess correlates of depressive symptom severity and predictors of having depressive disorder (PHQ-9), respectively, among household members.

**Results:**

We observed that roughly one in five household members (19%) screened positive for a depressive disorder (PHQ-9 > 9). More than half of household members endorsed six or more of the nine symptoms, with 68% reporting feeling ‘down, depressed, or hopeless’ in the prior two weeks. Elevated depression symptom severity was associated with greater disability (β = 0.17, *p* < 0.001), less social support (β = −0.04, *p* = 0.016), and lower self-reported overall health (β = 0.54, *p* = 0.001). Having depressive disorder was also associated with greater disability (adjusted Odds Ratio [aOR] = 1.12, *p* = 0.001) and less social support (aOR = 0.97, *p* = 0.024).

**Conclusions:**

In the Malawian context, we find that depressive disorder and depression symptoms are shared attributes among household members. This has implications for both screening and treatment, and it suggests that mental health should be approached from the vantage point of the broader social ecology of the household and family unit.

**Trial Registration:**

ClinicalTrials.gov (NCT04777006) - March 2, 2021.

## Background

Mental health conditions affect approximately one billion people around the globe [1]. One of the most common mental health conditions, major depressive disorder (MDD), represents the leading cause of disability worldwide [2]. In low-and-middle-income countries such as Malawi, MDD is even more prevalent in rural and remote areas where there are limited employment and income-generating opportunities [3,4]. Nevertheless, fewer than a quarter of those living with mental health conditions in low-and-middle-income countries receive treatment [5].

Individuals with chronic health conditions – such as Human Immunodeficiency Virus (HIV), diabetes, and hypertension – are more likely to be affected by mental health conditions [6,7]. For example, among people living with HIV, an estimated 39% have a depressive disorder [8]. An HIV diagnosis and the disease progression can lead to stress, social isolation, and comorbidities, which increase the risk of developing MDD [9,10]. Additionally, HIV medication side effects and neurological impacts from HIV are associated with depression symptoms [11]. Furthermore, people living with HIV who have comorbid MDD also have a lower likelihood of adhering to antiretroviral therapy (ART) and a higher likelihood of being virally unsuppressed [8,10].

Common forms of behavioral therapy that address MDD in high HIV burden settings include Problem-Solving Therapy [12], the Friendship Bench Model [13], and Interpersonal Therapy [14]. In each case, these treatments provide an array of tools that focus on enhancing coping mechanisms, promoting behavioral activation, and teaching cognitive restructuring, strategic problem-solving, and emotion regulation techniques. They also identify opportunities to reinforce social bonds – for example, by strengthening social skills and nurturing peer relationships.

Nevertheless, these therapeutic approaches have limited consideration for social-ecological factors likely to shape everyday environments – particularly household dynamics [15]. This shortcoming is notable for two reasons. First, in collectivist societies where HIV is most prevalent, household characteristics such as social, emotional, and financial support may exert an outsized influence on one’s disposition, at least in comparative terms to individualist societies [16]. Second, a growing body of evidence indicates social contagion characteristics of depression: interactions with family and friends who have a depressed mood or sense of hopelessness about the future can – over time – influence one’s own outlook [17].

Malawi represents a model collectivist setting for examining the interplay of household dynamics, mental health, and well-being. The country has one of the highest HIV incidence rates in the world [18] and an estimated point prevalence of depression of 19% [19]. While considerable resources and efforts have targeted the HIV epidemic, depression remains largely untreated [20,21]. Prior research has delineated the epidemiology of MDD in the Malawian context [22–24]. However, little is known about the relationship between individuals’ depressive symptoms and household characteristics.

This study aimed to investigate the household dynamics of adults living with MDD and other chronic health conditions (predominately HIV) in rural Malawi. Specifically, we investigated the prevalence and correlates of depressive symptoms and depressive disorder among household members of individuals with MDD and other chronic health conditions. The study is part of a randomized controlled trial that introduced and evaluated an evidence-based model of depression care at integrated chronic care clinic (IC3) facilities [25]. Household members provided written consent (including a thumbprint, if illiterate) and were interviewed prior to the initiation of depression care.

## Methods

### Setting and study design

This study is a part of a randomized controlled trial investigating an evidence-based model of depression care at IC3 in 14 health facilities throughout Neno District, Malawi. The IC3 paradigm represents an expansion of a care delivery system that was historically focused on HIV services alone. Since its inception, patients would travel to clinics on a quarterly basis to collect antiretroviral medications, creating a longitudinal pattern of care into which other chronic conditions could be integrated. Over the past eight years this has been broadened to include screening and treatment for a range of non-communicable diseases (NCDs) such as type-2 diabetes, hypertension, asthma, and epilepsy [26]. Neno District is a small, rural, Southern district of Malawi that has fully integrated chronic care into IC3. As of 2021, approximately one in every 10 individuals throughout Neno District – which comprises roughly 138,000 people – were enrolled in IC3, of which approximately 70% have a positive HIV diagnosis.

At baseline, 60% of individuals who met study inclusion criteria, including diagnosis of depression disorder (see below), were randomly selected to have a household member interviewed, pending the study member’s consent. This household interview, conducted in the month prior to the participant’s treatment initiation, examined clinical, health, and sociodemographic characteristics of household members in relation to the study participant; the study design is therefore cross-sectional. The study received institutional review board approval from the National Health Sciences Research Committee in Malawi and the RAND Corporation Institutional Review Board in the United States.

### Participants

At the stage of study participant recruitment, participants were asked if they would be open to a household member being interviewed, provided the household member was not informed of the study participant’s diagnosis. Of 487 study participants, 427 (88%) verbally consented to have a household member interviewed. Of the 427 consenting individuals, 258 were randomly selected for the household interview. Study participants were then asked to provide the name and (if available) contact information of an adult (age 18+) residing within their household and representing their primary social support, if they were not living alone.

One month prior to the initiation of the study member’s depression treatment, a data collector traveled to the individual’s household to meet with the household member. If the household member was unavailable during the visit, the data collector returned later. Out of the 258 household members identified by study participants, 237 were successfully contacted and 236 provided verbal consent to participate. Apart from residing in the same household as the study participant and being 18 years or older, there were no other inclusion or exclusion criteria.

Household participants were informed that the data collector was conducting a district-wide survey on household health and wellbeing throughout Neno District and that they were selected to participate. To protect confidentiality, household members were not informed of the depression diagnosis of those they were residing with, nor did we inform them that anyone else in their household was participating in a research study. The interview took approximately 30 minutes to complete, and participants were compensated with 2,500 Malawian Kwacha (roughly $3) for their time, an amount equivalent to opportunity costs.

### Measures

The household interview comprised six instruments, the first of which gathered an overview of the household member’s sociodemographic characteristics – such as sex, age, marital status, and source of income. The remaining instruments are described below.

Depressive symptoms and depressive disorder were evaluated using the nine-item version of the Patient Health Questionnaire (PHQ-9) [27], which has been previously translated and validated for use in Malawi [21]. The questionnaire asks about the frequency of depression symptoms over the past two weeks, on a four-point scale ranging from ‘never’ to ‘nearly every day’. A positive PHQ-9 score is an endorsement of depressive symptoms. Scores greater than nine and less than 15 indicate moderate depression, while those above 15 are considered severe depression.

Functional impairment was evaluated using the World Health Organization Disability Assessment Schedule, version 2.0 (WHODAS), which has been implemented in countries throughout sub-Saharan Africa, including Malawi [28]. It is a 12-item assessment covering types of disability ranging from difficulty with household responsibilities to maintaining friendships. The WHODAS covers disability over the past 30 days, placing severity on a four-point scale ranging from ‘none’ to ‘extreme/cannot do’.

Overall health was evaluated using the EuroQol, covering five dimensions of health at five levels (EQ-5D-5 L) [29]. The tool has been previously validated for use in Malawi and provides a continuous measure of health which can be translated into quality-adjusted life years (QALYs) [30,31]. It asks about individuals’ health today, and response options are on a five-point scale ranging from ‘no problems’ to ‘extreme/unable to do’.

Social support was assessed using the Multidimensional Scale of Perceived Social Support (MSPSS), previously adapted to the Malawian context [32,33]. The survey asks 12 questions about dimensions of social support from friends and family members, focusing on the individual’s present circumstances. Response options are on a seven-point scale ranging from ‘very strongly disagree’ to ‘very strongly agree’.

We evaluated the household burden of care using the Burden Assessment Scale (BAS), which has been locally adapted to the study context [34]. It contains 19 items that assess the potential impacts of caring for a family member with a mental health condition – such as reduced leisure time, financial problems, and worry. The survey asks about the previous six months, and items are standardized on a four-point scale ranging from ‘not at all’ to ‘a lot’.

We gathered data from all the same instruments for corresponding study participants from the trial, except for the BAS, which was only collected during household interviews.

### Statistical methods

Based on our sample size, the present study had 80% power to detect relationships between patient and household member measures that were ≥ 0.19. As a first step in analysis, we drew from baseline interviews to generate descriptive statistics that characterized patients and household members. This included frequencies for discrete variables and means and standard deviations for continuous variables. The chief interest in this study was the prevalence of depression symptoms (PHQ-9 score > 0) and the prevalence of depressive disorder (PHQ-9 score > 9) among household participants.

Next, we conducted multivariable linear regression to assess correlates of depressive symptom severity among household members, including health and sociodemographic characteristics as independent variables. We separately configured this analysis as a multivariable logistic regression, with the outcome structured as whether the household member had an indication of depressive disorder, based on a PHQ-9 score greater than nine (1=yes; 0=no).

## Results

### Sample characteristics

As shown in Table 1, household members were predominately female (61%) and between the ages of 18 and 30 years old (38%). The corresponding study participants were also predominately female (82%) and between the ages of 31 and 50 years old (61%). Half of the study participants were married (50%). The remainder were divorced (21%), single (15%), or widowed (14%). Corresponding household members were also predominately married (60%). Nearly nine in 10 household members reported having a source of income (86%). Almost an equal number reported some level of education (91%).

**Table 1.** Patient and household member characteristics.

Demographic Characteristic	Study Participants	Household Members
*Gender*		
Female (n)	82.2% (194)	61.0% (144)
Male (n)	17.8% (42)	39.0% (92)
*Age*		
18–30 (n)	8.1% (19)	38.1% (90)
31–40 (n)	29.7% (70)	21.2% (50)
41–50 (n)	31.4% (74)	12.7% (30)
51–60 (n)	19.1% (45)	14.8% (35)
61–70 (n)	7.2% (17)	6.4% (15)
71+ (n)	4.7% (11)	6.8% (16)
*Marital Status*		
Single (n)	15.3% (36)	19.5% (46)
Married (n)	5.4% (119)	60.2% (142)
Divorced (n)	2.8% (49)	10.6% (25)
Widowed (n)	13.6% (32)	9.7% (23)
*Any Source of Income*		
No (n)	36.4% (86)	14.0% (33)
Yes (n)	63.6% (150)	86.0% (203)
*Any Education*		
No (n)	14.0% (33)	9.3% (22)
Yes (n)	86.0% (203)	90.7% (214)
*Depression Severity*^a,b^		
Moderate (n)	73.3% (173)	16.1% (38)
Severe (n)	26.7% (63)	3.4% (8)
*Disability*		
Mean (SD) WHODAS^c^ (Scale: 0–45)	14.8 (8.0)	6.5 (6.9)
*Overall Health*		
Mean (SD) EQ-5D-5 L^d^ (Scale: 5–15)	9.7 (1.8)	7.3 (1.9)
*Social Support*		
Mean (SD) MSPSS^e^ (Scale: 12–84)	59.4 (16.9)	65.0 (13.8)
*Burden of Care*		
Mean (SD) BAS^f^ (Scale: 0–76)	na	19.3 (13.6)

^a^Depression severity is considered moderate for a PHQ-9 score between 10 and 14 and considered severe for a PHQ-9 score of 15 or greater.

^b^This does not add up to 100%, because it has been limited to household participants with a PHQ-9 score of 10 or greater.

^c^WHODAS is the WHO Disability Assessment Schedule 2.0.

^d^EQ-5D-5 L is the EUROQOL five dimensions five levels survey.

^e^MSPSS is a Multidimensional Scale of Perceived Social Support.

^f^BAS is the Burden of Assessment Scale – this is only measured for household members.

### Prevalence estimates

**Figure 1.**
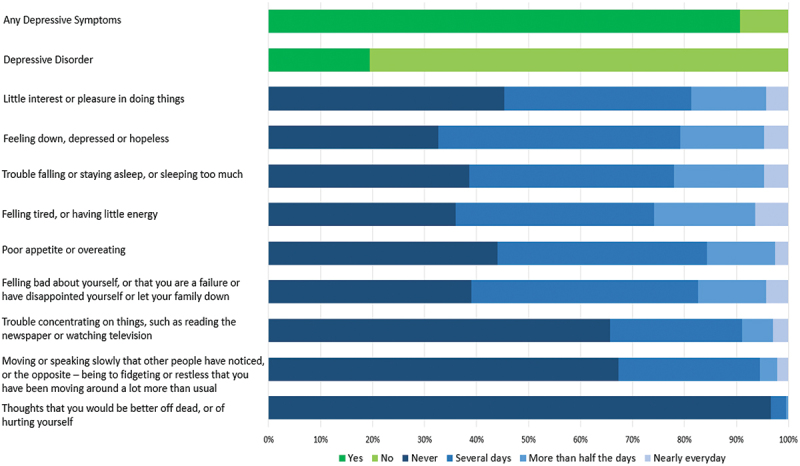
Prevalence of depressive symptoms and depressive disorder among household members.

Note: ‘Depressive Symptoms’ is defined as having a PHQ-9 score greater than 0. ‘Depressive Disorder’ is defined as having a PHQ-9 score of 10 or greater.

Overall, 16% of household members were classified as moderately depressed and three percent as severely depressed, based on PHQ-9 scores. This represents a 19% prevalence of depressive disorder. Greater than nine in 10 household members endorsed one or more depressive symptoms (see Figure 1) . The most frequently-endorsed item was: ‘feeling down, depressed or hopeless’ (several days: 47%; more than half the days: 16%; nearly every day: 5%). 64% of the respondents also reported ‘feeling tired or having little energy’, whilst 61% reported ‘feeling bad about yourself, or that you are a failure or have disappointed yourself or let your family down’.

### Correlates of depression

According to the results of the multivariable analysis (Table 2), elevated depression symptoms were associated with high levels of disability as measured by WHO-DAS (β = 0.17; standard error [SE] = 0.05; 95% confidence interval [CI] = 0.08, 0.26), lower overall health as measured by the EQ-5D-5 L (β = 0.54; SE = 0.16; 95%CI = 0.23,0.85), and weaker social support as measured by the MSPSS (β = −0.04; SE = 0.02; 95%CI = −0.07, −0.01). No other measures were significantly correlated with depression symptom severity (*p* > 0.05).

**Table 2.** Depression symptom severity and depressive disorder correlates.

Characteristic	Depression Symptom Severity		Depressive Disorder	
	Coefficient (95%CI)	*p* value	Adjusted Odds (95%CI)	*p* value
*Gender*				
Male	−0.86 (−1.85, 0.13)	0.088	1.00 (0.42, 2.36)	0.997
Female	*ref*		*ref*	
*Age*				
31–40	−0.59 (−1.82, 0.64)	0.344	0.94 (0.32, 2.74)	0.904
41–50	0.19 (−1.33, 1.72)	0.804	0.96 (0.24, 3.78)	0.953
51–60	0.22 (−1.28, 1.72)	0.774	1.53 (0.46, 5.07)	0.482
61–70	−0.65 (−2.78, 1.48)	0.549	0.83 (0.16, 4.35)	0.821
71+	−1.20 (−3.47, 1.06)	0.297	0.13 (0.02, 1.17)	0.069
18–30	*ref*		*ref*	
*Marital Status*				
Married	−0.27 (−1.77, 1.22)	0.719	0.50 (0.15, 1.70)	0.268
Single	−0.26 (−2.04, 1.53)	0.775	0.89 (0.21, 3.75)	0.875
Widowed	−0.33 (−2.47, 1.81)	0.760	1.23 (0.24, 6.27)	0.806
Divorced	*ref*		*ref*	
*Any Source of Income*				
Yes	−0.22 (−1.58, 1.14)	0.752	0.71 (0.25, 2.00)	0.516
No	*ref*		*ref*	
*Any Education*				
Yes	0.21 (−1.45, 1.87)	0.802	0.87 (0.23, 3.34)	0.838
No	*ref*		*ref*	
*Disability*				
WHODAS^a^ (Scale: 0–45)	0.17 (0.08, 0.26)	<0.001	1.12 (1.05, 1.20)	0.001
*Overall Health*				
EQ-5D-5 L^b^ (Scale: 5–15)	0.54 (0.23, 0.85)	0.001	1.24 (0.97, 1.59)	0.088
*Social Support*				
MSPSS^c^ (Scale: 12–84)	−0.04 (−0.07, −0.01)	0.016	0.97 (0.94, 1.00)	0.024
*Burden of Care*				
BAS^d^ (Scale: 0–76)	0.03 (−0.01, 0.07)	0.188	0.98 (0.94, 1.01)	0.156

^a^WHODAS is the WHO Disability Assessment Schedule 2.0.

^b^EQ-5D-5 L is the EUROQOL five dimensions five levels survey.

^c^MSPSS is a Multidimensional Scale of Perceived Social Support.

^d^BAS is the Burden of Assessment Scale.

Depressive disorder (PHQ-9 > 9) was associated with greater disability as measured by the WHO-DAS (adjusted odds ratio [aOR] = 1.12; SE = 0.04; 95%CI = 1.05, 1.20) and low social support as measured by the MSPSS (aOR = 0.97; SE = 0.01; 95%CI = 0.94,1,00). No other factors were significantly associated with having a depressive disorder (*p* > 0.05).

## Discussion

To our knowledge, this is one of the first studies to assess the prevalence and correlates of depressive symptoms and depressive disorder among household members of patients with MDD. We found a high prevalence of depressive symptoms: 91% of household respondents endorsed one or more symptoms, a figure similar to that observed by Docrat and colleagues (2019) among household members within a primary healthcare setting in South Africa [35]. Other studies, looking specifically at the prevalence of depression symptoms among caregivers, have reported lower rates that were nevertheless elevated compared to those found in the overall population [36,37].

The prevalence of depressive disorder among household members (19%) was higher than estimates reported for other sub-populations in Malawi [23] and comparable to other high-risk groups [38] within the Malawian context as well. In Ethiopia, Derajew and colleagues (2017) reported the same prevalence of depression (19%) among primary caregivers of household members previously diagnosed with depression [39]. Meanwhile, studies in China [40] and Malaysia [41] also looking at caregivers, have reported modestly lower depression prevalence estimates, though (again) higher than in the general public. Differences in estimates may be accounted for by the characteristics of study participants and tools selected for measuring depression. For instance, Chai and colleagues (2021) used a combination of depression assessment tools [41], while this study relied on the PHQ-9 instrument. However, all four studies (including ours) suggest that depression in one household member may serve as an important proxy for depression screening among all household members.

We also found that specific constructs were associated with greater or lesser depression severity. For example, household members who reported high levels of social support also reported lower levels of depression symptoms. This corroborates a previous study set in Malawi, which found the same pattern among caregivers of children in vulnerable households [37]. Similar findings have also been recorded in other countries [42,43]. Low levels of social support are associated with stress response mechanisms, particularly among those with caregiving responsibilities [44]. Prior studies have documented that increasing social support, either through formal mental health services or informally within the family unit, can alleviate depression symptoms [43,45,46]. Social support more generally also serves as a buffer and can be protective against the onset of depression [47].

Among household members, screening positive for depressive disorder was associated with greater functional impairment. Significant functional impairment and/or distress are requirements for the diagnosis of major depressive disorder according to the Diagnostic and Statistical Manual of Mental Disorders (DSM) and International Classification of Diseases (ICD) criteria [48]. As such, this finding is not surprising; however, it does provide concurrent validity to our study’s measurement of depression with the PHQ-9. Furthermore, it underscores that – in the Malawian context as in many other settings [49,50]—depression substantively shapes daily activities such as the completion of household chores and interactions with family and friends. Likewise, we observed that lower overall health, as reported by the EQ-5D-5 L, which includes caring for oneself and the ability to perform usual activities, was associated with elevated depression symptomology among household members. Similar findings on health related quality of life have been documented elsewhere [51,52]. Our study cannot infer whether deprecated overall health is a reflection of self-reported depression symptoms, depression symptoms affect physical health, physical health affects depression, or some combination.

In contrast with past studies, we do not find evidence that household members’ depressive symptoms and depression were related to demographic characteristics such as age, gender, marital status, or income [39,53,54]. Similarly, the relationship between depressive symptoms and the burden of care on the household member was not significant, which has been documented previously among caregivers of patients living with chronic health conditions [55,56]. One possibility is that our sample was relatively homogeneous with respect to certain characteristics (for example, disproportionately female, rural, low-income); coupled with a small sample size, we were underpowered to detect correlations less than 0.19.

Our study has several limitations. First, as just noted, study participants were relatively homogeneous. This reduces variance and limits our ability to generalize findings to other Malawian or similar regional settings. Second, unlike prior investigations, we did not restrict analyses to principal caregivers of the patients and instead extended eligibility to all adult household members. This determination was based, in part, on the age range of our sample. Third, all significant relationships reported in this study are correlational. To conclude directionality of relationships would require an experimental or quasi-experimental approach. Likewise, some relationships of interest (e.g. CD4 count) were unmeasurable due to resource limitations in the setting. Lastly, we quantified depressive symptoms using the PHQ-9. While the PHQ-9 has been validated for use in Malawi [21] complementary new methods that avoid forms of self-response bias might be considered in future research efforts [57].

## Conclusion

We found a high prevalence of depressive symptoms and depression among household members of chronic care patients with MDD. The overall paucity of resources presently available to households – coupled with difficulties in social support, overall health, and disability – make a collective case for the importance of interventions that address these complexities. While certain forms of aid may be unavailable in rural Malawi, depression care interventions could directly consider household dynamics, the role of social support and social networks in shaping trajectories, and even extend treatment to household members who screen positive for depressive disorder.

## Data Availability

Deidentified data and code are available upon request (email: rmcbain@bwh.harvard.edu).

## Paper context

Among household members of adults living with major depressive disorder, we find a high prevalence of depressive symptoms and moderate-to-severe depressive disorders. The findings shed new light on the social ecology of major depressive disorder, including the correspondence of elevated depressive symptoms among co-habiting household members. The findings indicate that mental health screening and treatment should extend beyond the individual perspective – to consider screenings among household members and interventions that account for household dynamics.
